# Farther Observations on the Action of Lime Water and Magnesia on Common Peruvian Bark

**Published:** 1787

**Authors:** Thomas Skeete

**Affiliations:** Physician to the New Finsbury Dispensary


### IX. *Farther Observations on the Action of Lime Water and Magnesia on common Peruvian Bark. By* Thomas Skeete, *M. D., Physician to the New Finsbury Dispensary.*

DR. Irving having, in the last volume* of the London Medical Journal, endeavoured to invalidate the conclusions which I have drawn on this subject in my Treatise on Bark, I shall now add a few remarks, which, I trust, will confirm my former opinions. I must observe, in the first place, that Dr. Irving has entirely mistaken my experiments, when he alledges, that, in comparing the weight of an infusion of bark in lime water with the common infusion, I had not attended to the weight of the lime. He will find, by referring to them with attention, that I have not been guilty of the inaccuracy with which he has charged me, the comparison having been first made between the infusion in lime water and lime water itself, and then the proportion of bark which had been dissolved compared with the increase of weight in the simple infusion. — Dr. Irving has also objected to the high red colour of the infusion of bark

* Vol. VII. page 419.

in lime water as a proof of its ftrength; and had I refted it on this or any other fingle circumftance, I might probably have failed in my attempt. He obferves, that he can at pleafure produce the colour by adding a portion of lime water to a fimple infufion; but that this, inftead of rendering the folution more complete, precipitates a quantity of the fufpended matter. He accounts, therefore, for the red colour of an infufion of bark in lime water from the want of that matter, the depofition of which from the fimple infufion produces the red colour. This matter, he thinks, is of a gummy nature, and capable of affecting the rays of light in fuch a manner as to produce the palenefs of the fimple infufion. Although this theory may at firft fight appear ingenious, it will be found, on examination, to be very inadequate to the purpofe; for if fome of the fal fodæ be added to an infufion of bark, it produces a deeper red colour than that which arifes from the addition of lime water, without the flighteft appearance of precipitation. The precipitation of a gummy matter being effential to the production of the colour, according to Dr. Irving, and the colour, as we have juft feen, being produced by the fal fodæ, without fuch precipitation, this this idea of the ſubject muſt neceſſarily be erroneous.

But that the colour is not entirely to be given up as one proof of the ſtrength of the infuſion of bark in lime water, or as a circumſtance accompanying other more ſatisfactory proofs, will appear from what follows:—If, for example, the colour of an infuſion of bark, carefully prepared with lime water, by rubbing them together, be compared with a mixture of lime water and infuſion of bark, a remarkable difference will be perceived; the firſt being of a fine bright red colour, and tranſparent; the laſt being only of a dirty reddiſh colour. The former poſſeſſes a ſtrong bitter aſtringent taſte; the latter is almoſt inſipid. The one is an efficacious remedy in the cure of ſeveral diſeaſes; the latter cannot be depended upon. The only concluſion therefore to be drawn from Dr. Irving's obſervations is, that the effects produced by bark and lime water are different, according to the manner and proportions in which they act upon each other; and this is what I have endeavoured to inculcate both with regard to lime and magneſia. To adminiſter bark and lime water with effect, we ſhould direct the lime water to be carefully rubbed with the the bark; avoiding the inelegant and comparatively inefficacious method of adding the lime water to an infufion or decoction of bark previoufly prepared.

The proof of the ftrength of the infufion of bark in lime water, deduced from the addition of the fal martis, I confefs, was in part erroneous, at leaft, I fhould have taken notice of the decompofition of the falt, by means of the lime, and the confequent formation of gypfum. I am ready to grant to Dr. Irving, that the precipitation partly depended on the gypfum, and on the calx of iron neceffarily depofited during the decompofition: but I cannot fo readily give up the proof arifing from the very dark or black colour of the precipitate, this being a ftrong evidence of the greater degree of aftringency in fuch an infufion. Dr. Irving, indeed, affirms, that by lime water alone, added to a folution of fal martis, he can obtain a ferrugineous precipitate, equally black with that which I have defcribed. I find, however, on making the comparifon, that the precipitate from fal martis, by means of lime water, is of a fine blue colour; whereas, the precipitate from an infufion of bark, prepared with lime water, is very dark coloured, or black: this circumſtance, therefore, muſt ſtill be admitted as one proof of the ſtrength of ſuch a preparation. The marks of aſtringency were ſtill more obvious in the preparation of bark with magneſia; for I have mentioned in page 28 of my treatiſe, that when a ſolution of ſal martis was added to an infuſion of this kind, a very deep black colour was produced.

As my purſuits do not permit me to enter into a minute detail on this, or any other chemical ſubject, I ſhall reſt ſatisfied with what I have already advanced, eſpecially as the good opinion I entertain of the preparations of bark in queſtion, is not ſupported by chemical proofs only, but by their effects on the body, which I conſider as more ſatisfactory. Dr. Irving ſeems to rely entirely on the chemical properties, which will frequently be found fallacious, the greateſt accuracy and chemical knowledge being ſometimes inſufficient to guard againſt the accidents which tend to alter the reſult of ſuch nice experiments. At the time that I publiſhed my obſervations on bark, I aſſerted the efficacy of the preparation with lime water without heſitation, as I had frequently ſeen it uſed, and had received the beſt accounts of it from ſeveral practitioners who had long employed ployed it. I could not then ſpeak with equal certainty of the preparation with magneſia, as many ſuccefsful caſes are required to eſtabliſh a remedy of this kind, nor have I yet had ſuffi-cient experience with regard to it. Such trials, however, as I have made with it, have tended to confirm the good opinion which I formed of it, from its ſenſible qualities and chemical properties. When well prepared, it is certainly a very elegant medicine. Its beautiful colour and tranſparency, and the length of time it will remain free from fermentation, are very ſtriking advantages, and will, I truſt, bring it at length into general uſe.

The following is the formula which I commonly employ.

℞. Pulv. cort. Peruv. ℥ſs.

Magneſ. alb. calcinat. ʒj.

Tere ſimul probe ad quartam horæ partem cum pauxillo aq. pur. ut fiat paſta; dein adde paulatim aq. pur. ℥ix. Infunde poſtea per ſemi-horam, vaſe ſubinde agitato, & liquorem per chartam cola.

The whole of the above quantity may be conveniently taken in a day, in divided doſes of of three or four table ſpoonfuls, in the ſame manner as the common infuſion of bark.

